# Alleviation of Ultraviolet B-Induced Photodamage by *Coffea arabica* Extract in Human Skin Fibroblasts and Hairless Mouse Skin

**DOI:** 10.3390/ijms18040782

**Published:** 2017-04-07

**Authors:** Po-Yuan Wu, Chi-Chang Huang, Yin Chu, Ya-Han Huang, Ping Lin, Yu-Han Liu, Kuo-Ching Wen, Chien-Yih Lin, Mei-Chich Hsu, Hsiu-Mei Chiang

**Affiliations:** 1 Department of Dermatology, China Medical University Hospital, Taichung 40402, Taiwan; wu.poyuan@gmail.com; 2 School of Medicine, China Medical University, Taichung 404, Taiwan; 3 Graduate Institute of Sports Science, National Taiwan Sport University, Taoyuan 33301, Taiwan; john5523@ntsu.edu.tw; 4 Department of Cosmeceutics, China Medical University, Taichung 40402, Taiwan; dooo517@outlook.com (Y.C.); hedy9088723@hotmail.com (Y.-H.H.); a50535l@hotmail.com (P.L.); lesley456987@yahoo.com.tw (Y.-H.L.); kcwen0520@mail.cmu.edu.tw (K.-C.W.); 5 Department of Biotechnology, Asia University, Taichung 41354, Taiwan; yihlin@asia.edu.tw; 6 Department of Sports Medicine, Kaohsiung Medical University, Kaohsiung 80708, Taiwan; meichich@kmu.edu.tw

**Keywords:** *Coffea arabica*, photodamage, antioxidant, inflammatory, nuclear factor-kappa B (NF-κB), inhibitor κB (IκB)

## Abstract

*Coffea arabica* extract (CAE) containing 48.3 ± 0.4 mg/g of chlorogenic acid and a trace amount of caffeic acid was found to alleviate photoaging activity in human skin fibroblasts. In this study, polyphenol-rich CAE was investigated for its antioxidant and antiinflammatory properties, as well as for its capability to alleviate ultraviolet B (UVB)-induced photodamage in BALB/c hairless mice. The results indicated that 500 μg/mL of CAE exhibited a reducing power of 94.7%, ferrous ion chelating activity of 46.4%, and hydroxyl radical scavenging activity of 20.3%. The CAE dose dependently reduced UVB-induced reactive oxygen species (ROS) generation in fibroblasts. Furthermore, CAE inhibited the UVB-induced expression of cyclooxygenase-2 and *p*-inhibitor κB, and the translocation of nuclear factor-kappa B (NF-κB) to the nucleus of fibroblasts. In addition, CAE alleviated UVB-induced photoaging and photodamage in BALB/c hairless mice by restoring the collagen content and reduced UVB-induced epidermal hyperplasia. CAE also inhibited UVB-induced NF-κB, interleukin-6, and matrix metalloproteinase-1 expression in the hairless mouse skin. The results indicated that CAE exhibits antiphotodamage activity by inhibiting UV-induced oxidative stress and inflammation. Therefore, CAE is a candidate for use in antioxidant, antiinflammatory, and antiphotodamage products.

## 1. Introduction

In recent years, the incidence of skin cancer has increased, possibly because of the depletion of the ozone layer, causing an increased exposure to solar radiation, and the use of ultraviolet (UV) tanning beds [1]. Skin aging depends on intrinsic factors, such as genetics and hormones, and extrinsic factors, such as UV radiation and environmental pollutants. UV radiation is the most crucial factor for skin aging, or photoaging, which is characterized by wrinkling, rough skin, and hyperpigmentation [2,3]. UVB irradiation stimulates inflammation and reactive oxygen species (ROS) formation, promoting aging-related signal transduction resulting in skin damage and photoaging. Collagen is one of the components of the extracellular matrix, which is synthesized by fibroblasts in the dermis, and contributes to skin strength and resilience [4,5].

UV irradiation produces peroxynitrite and ROS [6]. ROS upregulate cyclooxygenase (COX)-2 expression to stimulate inflammation, which then cause skin erythema and sunburn [7,8,9]. UV exposure generates prostaglandin E2 and leukotrienes that lead to erythema, edema, and inflammation [10]. Under normal conditions, nuclear factor-kappa B (NF-κB) binds to inhibitor κB (IκB) and forms an inactive complex in the cytoplasm [11,12]. When stimulated by UV irradiation, the ubiquitination of IκB triggers the translocation of NF-κB into the nucleus, which further increases the production of matrix metalloproteinase (MMP)-1, and subsequently, the degradation of collagen [12,13,14]. In addition, interleukins (ILs) and nuclear factor-kappa B (NF-κB) may induce COX-2 expression in the skin, causing skin photodamage [15].

Recently, increasing attention has been focused on the development of natural or plant products for use as antioxidants, antiinflammatory agents, and immunomodulatory agents, to prevent age-related disorders [16,17,18,19]. The application of antioxidants to the skin surface protects the skin from oxidative damage and photodamage for a long time [19,20,21]. Many plant antioxidants also exhibit antiinflammatory activity and can be used to alleviate oxidative stress-induced damage, such as photodamage and skin cancer [22,23,24,25,26]. Coffee is one of the most popular beverages in the world and a major source of dietary antioxidants; it has also been suggested to inhibit inflammation and scavenge free radicals [27,28]. Furthermore, coffee bean extract inhibits the formation of lipid peroxide and superoxide free radicals [29,30]. In a clinical study, a cream prepared from coffee berry extract was observed to reduce wrinkles and hyperpigmentation of the human skin, and improve the skin appearance [31]. In addition, coffee berry extract upregulated the gene and protein levels of collagens and growth factors, and downregulated the gene expression of MMPs in human cultured fibroblasts [31]. In our previous study, *Coffea arabica* extract (CAE) obtained from the leaves of *C. arabica* showed high 1,1-diphenyl-2-picrylhydrazyl (DPPH) radical scavenging activity and prevented 2,2′-azobis (2-amidinopropane) dihydrochloride (AAPH) radical-induced hemolysis of erythrocytes [32]. Furthermore, CAE promoted type I procollagen expression, suppressed MMP expression, and inhibited MAP kinase activation in human foreskin fibroblast (Hs68) cells [32]. Our previous study and the aforementioned studies indicate that CAE is a candidate for use in antiaging and antiphotoaging products.

This study investigated the potential of CAE to counteract UVB irradiation-induced oxidative stress, inflammation, and photodamage in fibroblasts and hairless mice, and elucidated the associated mechanisms.

## 2. Results

### 2.1. Antioxidant Activity of Coffea arabica Extract (CAE)

**Figure 1:**
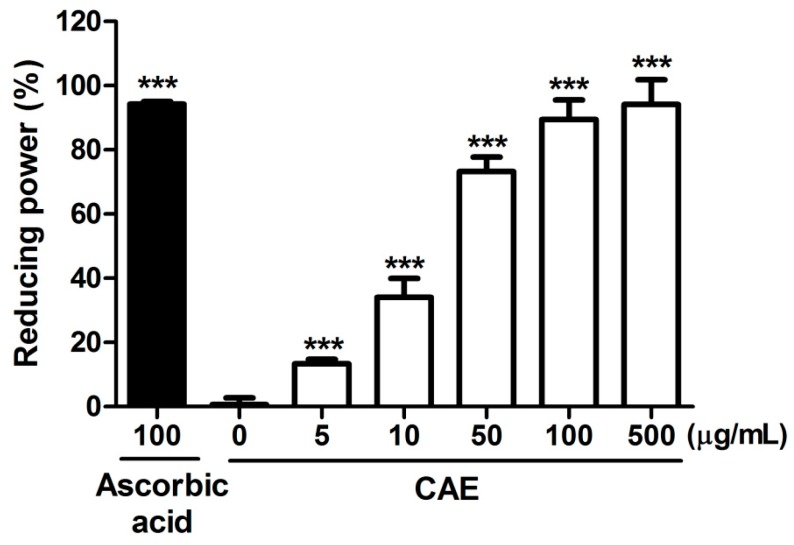
Reducing capability of CAE. Ascorbic acid (100 μg/mL) was used as the positive control. The reducing capability of CAE at concentrations above 100 μg/mL was similar to that of ascorbic acid. Significant difference versus control in each experiment: ***, *p* < 0.001.

**Figure 2:**
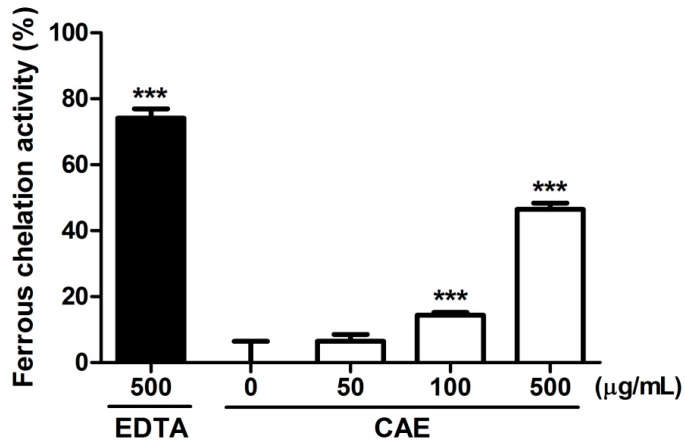
Ferrous ion chelating activity of CAE. EDTA (500 μg/mL) was applied as the positive control. A significant difference between this activity and that of the untreated (0 μg/mL CAE) group could be observed. Significant difference versus control in each experiment: ***, *p* < 0.001.

The reducing capability of CAE and ascorbic acid (positive control) observed in the present study is shown in Figure 1. CAE exhibited a potent reducing activity, and the reducing activity of 50 μg/mL of CAE was 80%. Furthermore, the reducing activity of 100 μg/mL of CAE was similar to that of ascorbic acid at the same concentration. Figure 2 shows the chelating activity of CAE and EDTA (500 μg/mL, the positive control). The metal chelating activity of 500 μg/mL of CAE was 46.4% ± 2.0% and that of EDTA at the same concentration was 74.5% ± 3.1%.

**Figure 3:**
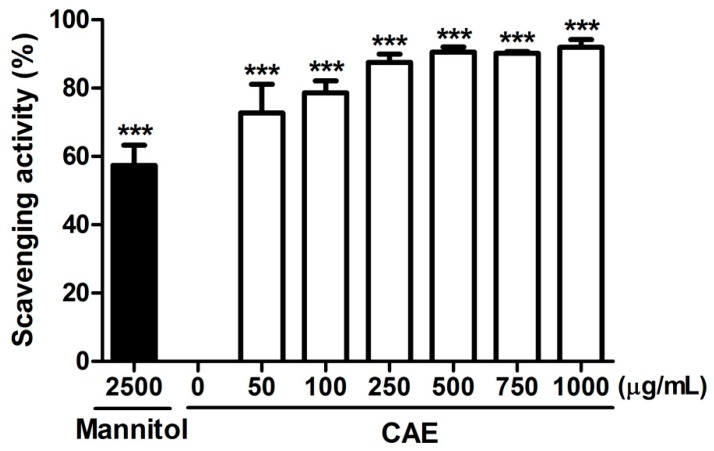
Hydroxyl radical scavenging activity (%) of CAE. Mannitol (2500 μg/mL) was used as the positive control. CAE exhibited powerful hydroxyl radical scavenging activity. Significant difference versus control in each experiment: ***, *p* < 0.001.

**Figure 4:**
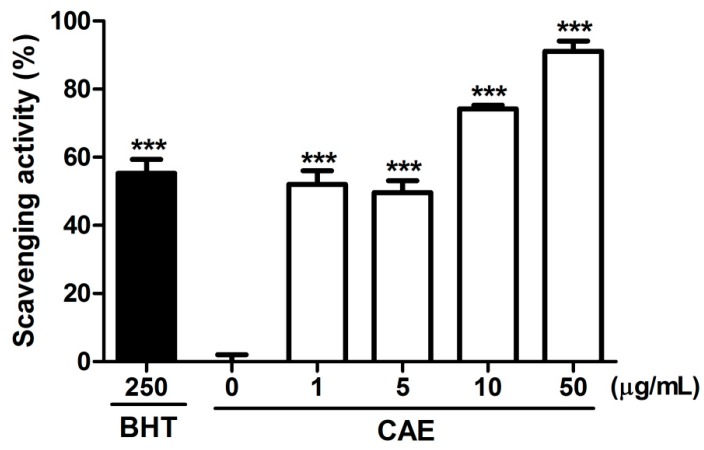
Superoxide anion scavenging activity (%) of CAE. BHT (250 μg/mL) was used as the positive control. The superoxide anion scavenging activity of 1 and 5 μg/mL CAE was similar to that of BHT. Significant difference versus control in each experiment: ***, *p* < 0.001.

**Figure 5:**
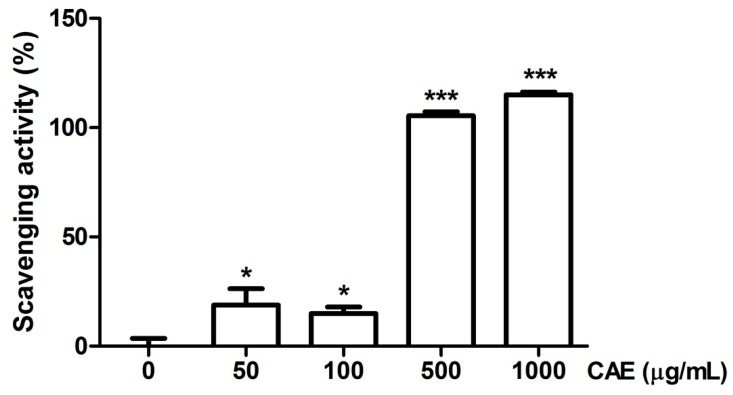
Hydrogen peroxide scavenging activity (%) of CAE. A significant difference between this activity and that of the untreated (0 μg/mL CAE) group can be observed. Significant difference versus control in each experiment: *, *p* < 0.05, ***, *p* < 0.001.

The scavenging capability of CAE was evaluated for various free radicals. The hydroxyl radical scavenging activity of CAE and the positive control (mannitol) is presented in Figure 3. The hydroxyl radical scavenging activity of 50 μg/mL of CAE was 72.7% ± 6.6%, and that of 2500 μg/mL of mannitol was 57.4% ± 4.8%. The superoxide anion scavenging activity of CAE is shown in Figure 4. The superoxide anion scavenging activity of dibutyl hydroxy toluene (BHT, positive control) was 55.9% ± 6.7%. The superoxide anion scavenging activity of 1 μg/mL of CAE was similar to that of 250 μg/mL of BHT (Figure 4). In addition, the peroxide scavenging activity of CAE is shown in Figure 5. The peroxide scavenging activity of 50, 100, 500, and 1000 μg/mL of CAE was 18.8% ± 7.5%, 14.9% ± 3.0%, 105.4% ± 1.9%, and 115.0% ± 1.2%, respectively. These results indicate that CAE exhibits a potent free radical scavenging activity.

### 2.2. CAE Inhibited ROS Generation in Human Skin Fibroblasts

**Figure 6:**
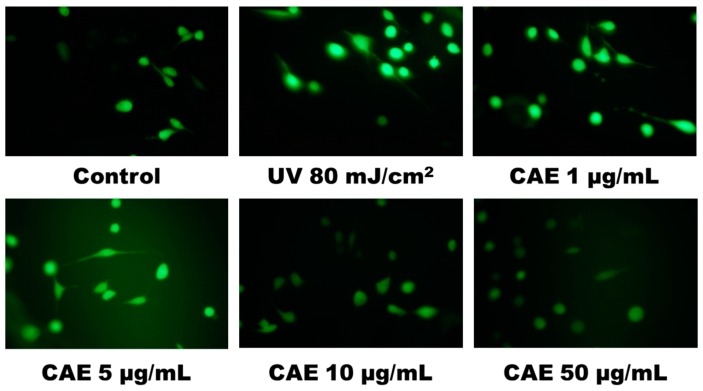
Examination of CAE inhibition of intracellular ROS generation in human skin fibroblasts by using a DCFH-DA assay (magnification factor: 200×). UVB irradiation increased ROS generation, whereas CAE inhibited ROS generation.

**Figure 7:**
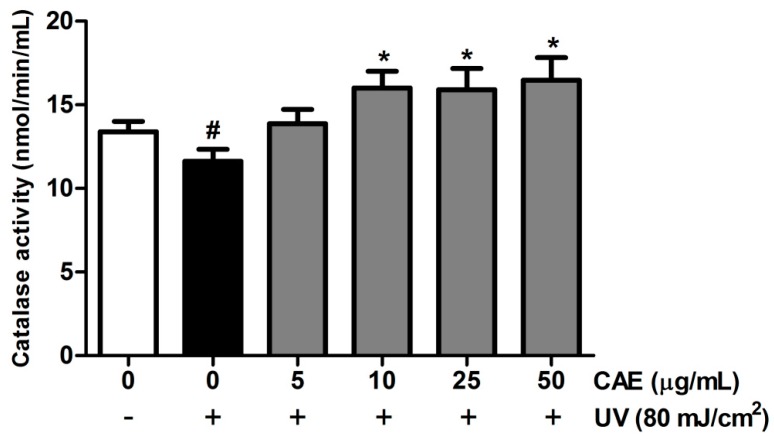
Effects of CAE on CAT activity in human skin fibroblasts. UVB irradiation inhibited CAT activity, whereas CAE treatment reversed the effect. Significant difference versus control: #, *p* < 0.05; significant difference versus the UVB-induced group: *, *p* < 0.05.

UV-induced ROS generation plays a crucial role in the photoaging of skin [33]. The intracellular ROS generation was assayed through 2′,7′-dichlorofluorescein diacetate (DCFDA) staining and fluorescence microscopy. Various CAE concentrations (1–50 μg/mL) dose dependently inhibited ROS generation in fibroblasts (Figure 6). In addition, UV irradiation inhibited catalase (CAT) activity, and postirradiation treatment with CAE enhanced CAT activity in human skin fibroblasts (Figure 7).

### 2.3. Effect of CAE on UVB-Induced Inflammation

**Figure 8:**
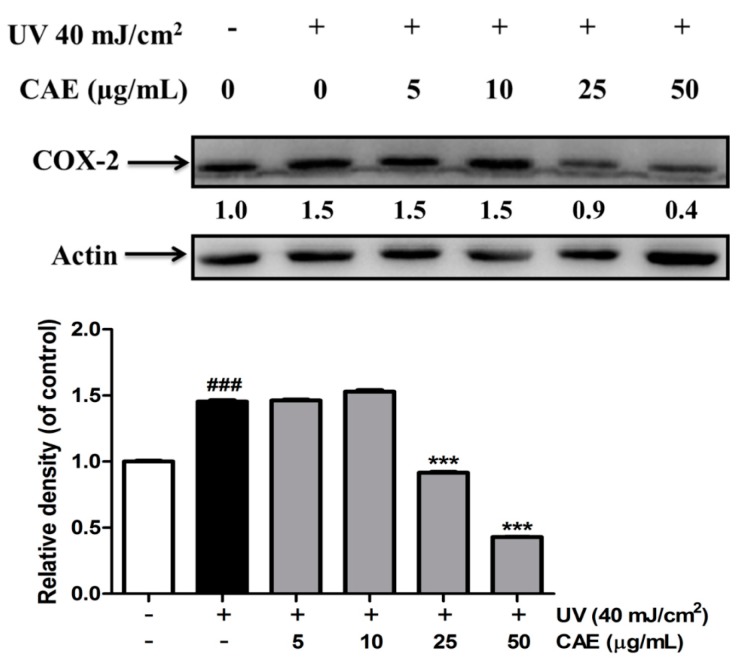
Effects of CAE on UVB-induced COX-2 expression in human skin fibroblasts. Significant difference versus control: ###, *p* < 0.001; significant difference versus the UVB-induced group: ***, *p* < 0.001.

UV-induced inflammation contributes to the chronological and premature aging of the skin [34]. The results of this study showed that the COX-2 level in UVB-exposed fibroblasts was 1.5-fold compared with that in the control cells (Figure 8). CAE treatment (5–50 μg/mL) inhibited the UVB-induced upregulation of COX-2, and this effect was substantial when the concentration of CAE exceeded 25 μM (Figure 8).

**Figure 9:**
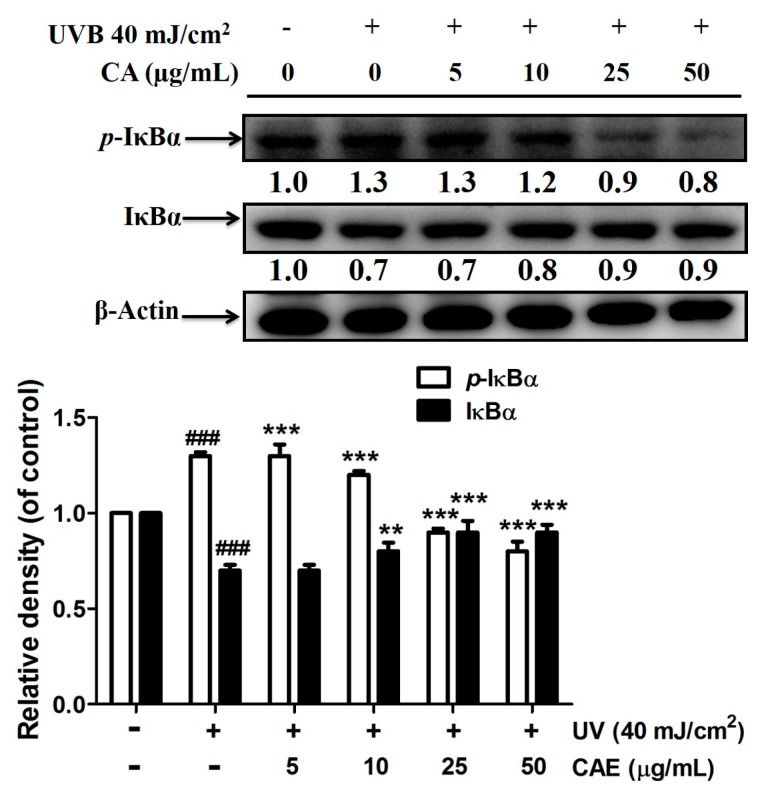
Effect of CAE on UVB-mediated *p*-IκBα and IκBα expression in human skin fibroblasts. Significant difference versus control: ###, *p* < 0.001; significant difference versus the UVB-induced group: **, *p* < 0.01, ***, *p* < 0.001.

**Figure 10:**
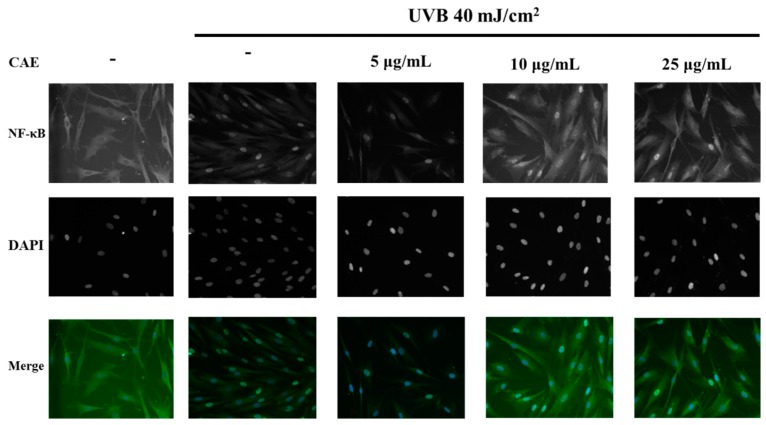
Effects of CAE on UVB-induced activation of NF-κB in human skin fibroblasts (magnification factor: 200×). UVB promoted the translocation of NF-κB in the nucleus and CAE inhibited this effect.

As shown in Figure 9, *p*-IκBα expression increased by 1.3-fold after UVB exposure, and CAE treatment considerably reduced *p*-IκBα expression. In addition, CAE considerably increased the IκBα level (from 0.7- to 0.9-fold; Figure 9). In this study, the NF-κB immunofluorescence staining assay in fibroblast cells was applied, to determine the level of NF-κB activation. Figure 10 shows that the translocation of NF-κB from the cytoplasm to the nucleus increased after UVB irradiation, whereas the translocation was inhibited after CAE treatment. The results of the immunofluorescence staining assay were consistent with IκB/*p*-IκBα expression. The results of the immunoblot analysis indicated that the protein level of IκBα was inhibited by ubiquitination following UVB irradiation, whereas *p*-IκBα expression was elevated (Figure 9). Subsequently, NF-κB translocated to the nucleus, causing an inflammatory response. However, CAE treatment inhibited the UVB-activated IκB/NF-κB signaling cascade, to ameliorate the inflammatory response.

### 2.4. Antiphotoaging Activity of CAE on UVB-Irradiated Hairless Mouse Skin

**Figure 11:**
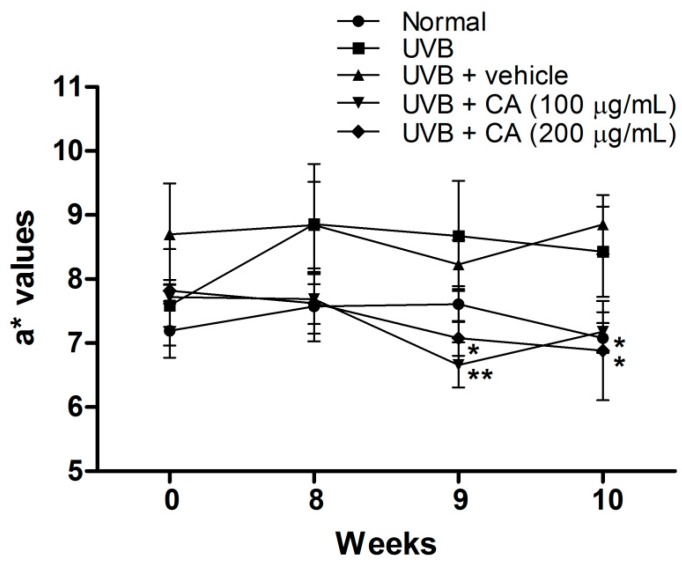
Effects of CAE on a* values in UVB-irradiated hairless mice in the tenth week. Significant difference versus the UVB-induced group: *, *p* < 0.05, **, *p* < 0.01.

After the 10-week experiment, the body weights of mice did not differ substantially in all the groups (data not shown). The a* value is an index of erythema and shows the inflammation degree in the skin. The a* values increased after UVB treatment, indicating that UVB induced erythema and inflammation of the skin (Figure 11). CAE treatment considerably reduced the a* values of the skin detected in the ninth and tenth week, and the a* values of CAE were similar to those of the normal group in the tenth week. These results suggest that CAE ameliorated UVB-induced inflammation and erythema.

**Figure 12:**
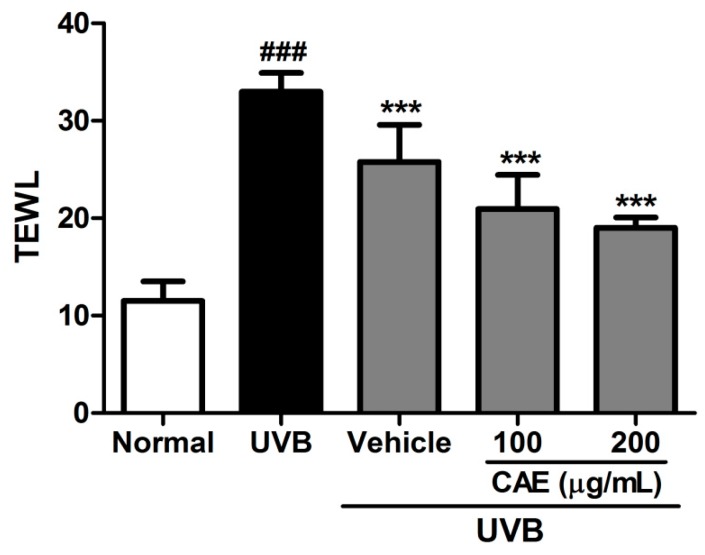
Effects of CAE on TEWL in chronic UVB-irradiated hairless mice. Significant difference versus control: ###, *p* < 0.001; significant difference versus the UVB-induced group: ***, *p* < 0.001.

The measurements of transepidermal water loss (TEWL) are useful for understanding the skin damage caused by some chemicals, pathological conditions, or environmental conditions, because the TEWL rate increases in proportion to the level of skin damage [35]. In the current study, UVB exposure of mice considerably enhanced the TEWL. The TEWL decreased after CAE application to hairless mice for 10 weeks (Figure 12), indicating that CAE did not cause skin toxicity or damage. In contrast, CAE appeared to protect the skin.

**Figure 13:**
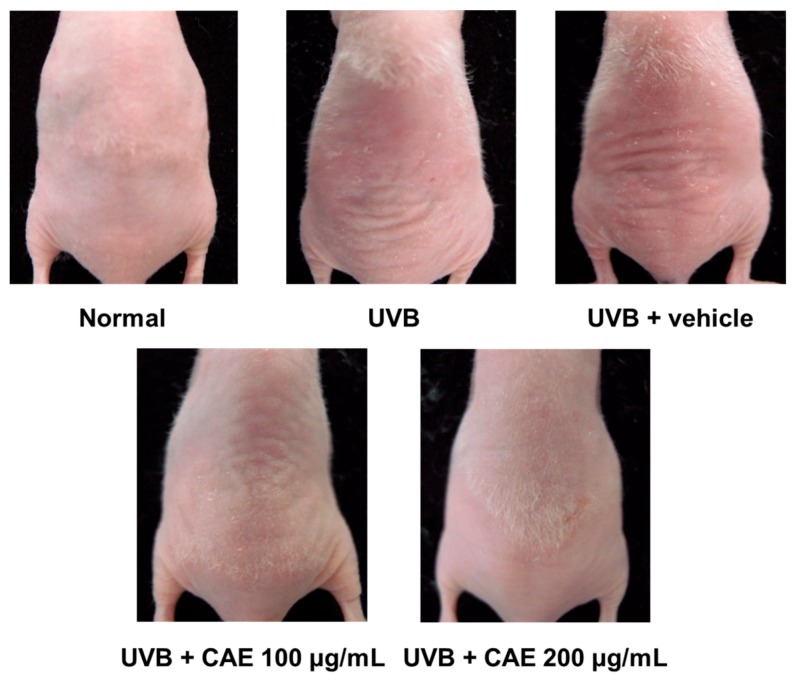
Photographs of wrinkles induced by UVB irradiation and the effect of topical application of CAE.

As shown in Figure 13, the formation of wrinkles was observed in the dorsal region in the UVB-exposed group. The topical application of CAE (100 and 200 μg/mL) reduced the wrinkle formation (Figure 13).

**Figure 14:**
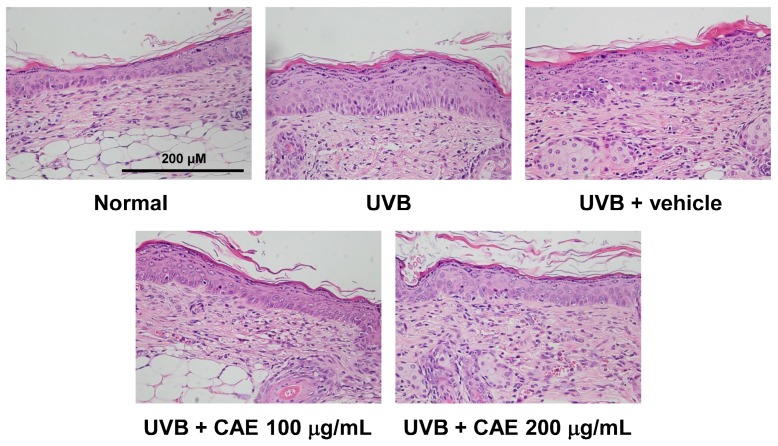
Light micrographs of the histological sections of hairless mice stained with H & E.

**Figure 15:**
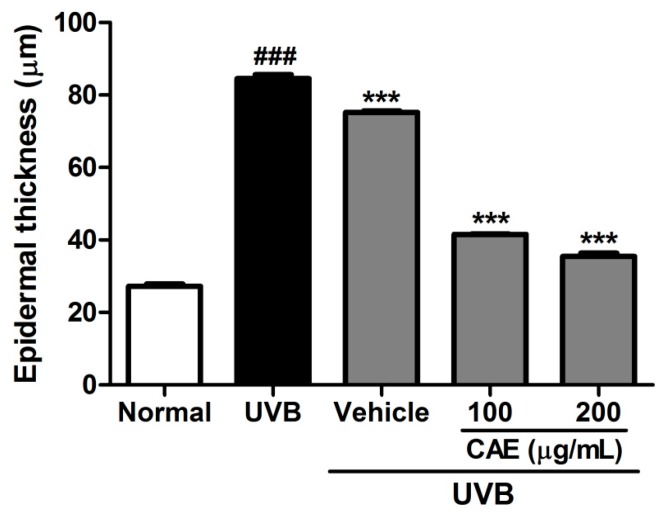
Skin thickness of hairless mice after CAE treatment. Significant difference versus the normal group: ###, *p* < 0.001; significant difference versus the UVB-induced group: ***, *p* < 0.001.

**Figure 16:**
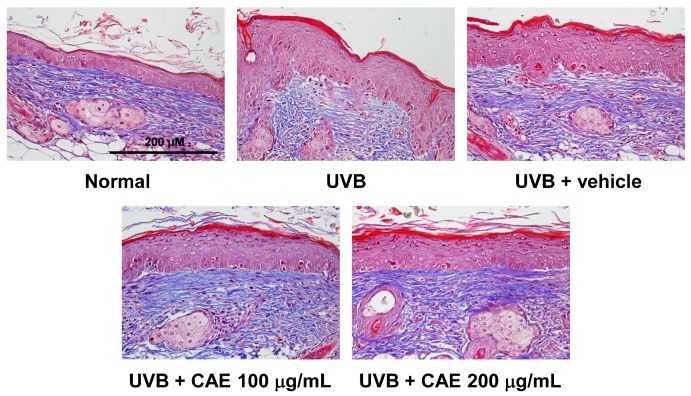
Light micrographs of histological sections of hairless mice stained with Masson’s trichrome. Collagen fibers were stained blue. UVB exposure reduced the collagen content in the mouse skin dermis and CAE treatment restored the collagen content.

The activity of CAE on the lesions, thickness, and collagen density of the skin in hairless mice after UVB irradiation was determined through histological examination. As shown in Figure 14 and Figure 15, the epidermal thicknesses of the UVB-exposed group, 100 μg/mL CAE-treated group, and 200 μg/mL CAE-treated group, were 84.6 ± 1.0, 41.5 ± 0.2, and 35.4 ± 0.9 μm, respectively. UVB irradiation resulted in a significant increase in the skin thickness compared with that of normal mice; however, the increase in the epidermal thickness caused by UVB irradiation decreased significantly after the topical application of CAE (Figure 14 and Figure 15). Masson’s trichrome staining of the dorsal skin showed that the collagen content decreased considerably after UVB exposure. The topical application of CAE increased the collagen amount in the dermis of BALB/c hairless mice (Figure 16).

### 2.5. CAE Inhibited MMP-1, iNOS, IL-6, and NF-κB Levels in UVB-Exposed Hairless Mouse Dermis

**Figure 17:**
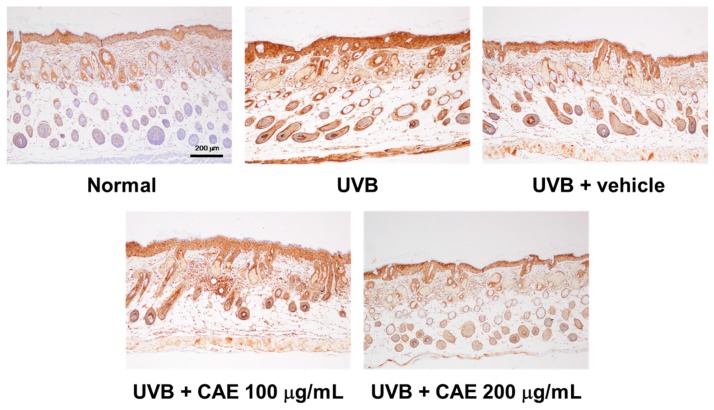
Immunohistological staining for MMP-1 in the hairless mouse skin after UVB exposure and CAE treatment. MMP-1 expression increased after UVB irradiation in the mouse skin, whereas CAE treatment inhibited the effect.

UVB-induced MMP-1 overexpression causes collagen degradation in the skin. As presented in Figure 17, MMP-1 expression increased after UVB irradiation, and CAE treatment reduced MMP-1 expression.

**Figure 18:**
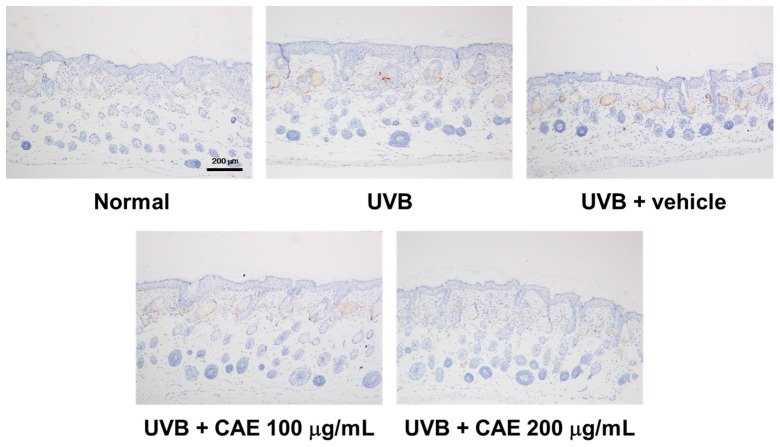
Immunohistological staining for iNOS in the hairless mouse skin after UVB exposure and CAE treatment.

**Figure 19:**
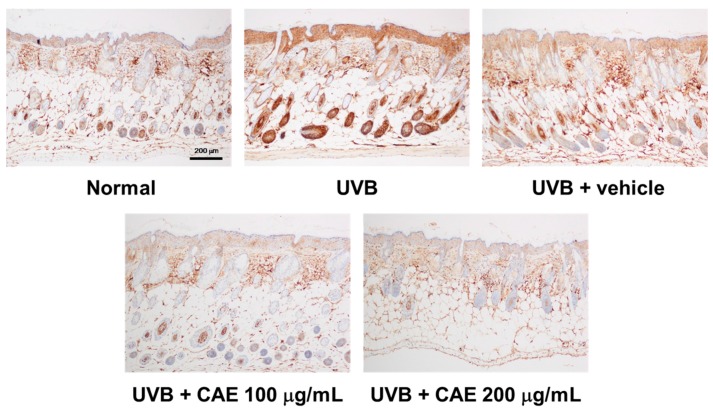
Immunohistological staining for IL-6 in the hairless mouse skin after UVB exposure and CAE treatment.

**Figure 20:**
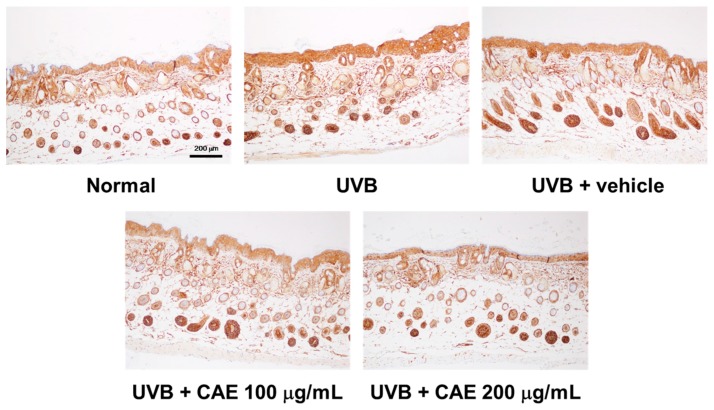
Immunohistological staining for NF-κB in the hairless mouse skin after UVB exposure and CAE treatment.

UVB upregulates inflammatory cytokines and the related factors, causing skin damage. Figure 18, Figure 19 and Figure 20 illustrate that UVB irradiation induced iNOS, IL-6, and NF-κB expression in the mouse skin, whereas the topical application of CAE significantly inhibited UVB-induced inflammation.

## 3. Discussion

Oxidation is the major cause of aging, and the biochemical reaction associated with the normal metabolic process often produces free radicals and ROS. Transition metal ions, including Fe^2+^ and Cu^2+^, participate in various oxidation reactions and catalyze hydroxyl radical formation [36]. Hydroxyl radicals are the most active ROS and rapidly react with biomembranes and biomolecules, causing severe damage. Metal ion chelation indirectly contributes to oxidative stress and ameliorates the process of skin aging. Ferrous chelation exhibits crucial antioxidant activity by inhibiting metal-catalyzed oxidation. In recent decades, natural products and plants have gained attention as photoprotective agents and may be added to skin care products for topical applications [19]. In our previous study, CAE with a rich polyphenol content exhibited DPPH scavenging activity and prevented erythrocytes from hemolysis induced by AAPH [32]. The results of this study revealed that the reducing power of CAE is similar to that of ascorbic acid. CAE chelated 46.4% of free Fe^2+^ and exhibited high scavenging activity for hydroxyl radicals, superoxide, and peroxide. Furthermore, the results of the free radical scavenging assays performed in this study indicated that CAE is a powerful antioxidant. CAE contains abundant polyphenols that can quench lipid peroxyl radicals and chelate iron in fibroblasts, thus inhibiting the initiation of lipid peroxidation [37]. Coffee bean extract scavenges free radicals and inhibits lipoxygenase activity [38]. The results reported in the present study are consistent with those reported in the literature.

A high concentration of ROS triggered by UV irradiation can cause various aging-related disorders, such as atherosclerosis, skin aging, and cancer [39]. UV exposure can deplete the endogenous oxidative stress defense system, resulting in ROS accumulation that can induce lipid peroxidation and macromolecule damage, especially in the skin. In addition, UV irradiation increases ROS generation and alters the functions and structures of proteins and genes, resulting in skin damage [2,33,40]. CAE, which inhibits ROS formation by UVB and acts as a UV absorber, can be used in photoprotective agents. CAE also increases CAT activity, which is depleted by UVB. UV irradiation activates MMPs to promote degradation and reduce collagen production in the skin, resulting in wrinkle formation and sagging skin [41,42]. CAE treatment can block UVB-induced collagen degradation in human skin fibroblasts [32]. The topical application of natural products with high antioxidant and antiinflammatory activity may ameliorate UV-induced photoaging [23,43]. In a previous study, the topical application of a cream containing coffee berry extract reduced wrinkle formation on the skin [31]. In addition, coffee berry extract inhibited MMP expression and stimulated collagen and growth factor levels [31]. The results of this study demonstrate that coffee leaf extract exhibits potent antioxidant activity and reduces wrinkle formation after UVB exposure.

Studies have demonstrated that the levels and expressions of proinflammatory factors, such as COX-2 and ILs, are induced by UVB exposure [7,9]. In the current study, CAE reduced UVB-induced COX-2 expression in fibroblasts and the hairless mouse skin, preventing the skin from photodamage. NF-κB/p65 is regulated by the phosphorylation of MAP kinases [44]. The upregulated ILs and NF-κB may stimulate the activity of COX-2 in keratinocytes and fibroblasts in the aged skin [15]. In our previous study, CAE treatment inhibited the phosphorylation of MAP kinases [32], which may inhibit the activation of NF-κB. NF-κB is bound to IκBs, a family of inhibitory proteins, and is present in the cytoplasm. The phosphorylation and degradation of IκB results in the translocation of NF-κB to the nucleus, which induces the transcription of proinflammatory mediators such as iNOS, ILs, and COX-2 [45]. The results indicated that CAE reduced the UVB-induced activation and translocation of NF-κB by IκBα protein degradation in fibroblasts. Coffee consumption is related to a lower risk of mortality and cardiovascular disease because of the antiinflammatory activity of coffee [27].

*C. arabica* contains chlorogenic acid, caffeine, cafestol, and caffeic acid [32,46,47]. The results of our previous study indicated that the total phenolic content of CAE was 26.7 ± 1.6 μg/mg, and CAE contained 48.3 ± 0.4 mg/g of chlorogenic acid and a small amount of caffeic acid [32]. Chlorogenic acid could inhibit the activation of inflammatory factors, such as COX-2, NF-κB, and iNOS, in the mouse skin after 12-*O*-tetradecanoylphorbol-13-acetate treatment and prevent skin cancer [48]. In addition, chlorogenic acid protects humans from the oxidative damage of macromolecules such as proteins and lipids [49]. Caffeic acid inhibited UVB-induced MMP-1 and -9 expression, and chlorogenic acid suppressed MMP-1 and -3 overexpression [32]. Therefore, polyphenols, chlorogenic acid, and caffeic acid may contribute to the antiinflammatory and antiphotodamage activity of CAE.

## 4. Materials and Methods

### 4.1. Chemicals and Materials

CAE with 48.3 ± 0.4 mg/g of chlorogenic acid and a trace amount of caffeic acid was prepared as described in a previous study [32]. The Bradford reagent for protein quantitation was obtained from Bio-Rad Laboratories (Berkeley, CA, USA). Fetal bovine serum (FBS), Dulbecco’s modified Eagle’s medium (DMEM), trypsin-ethylenediaminetetraacetic acid, and reagents for cell culture were purchased from Gibco, Invitrogen (Carlsbad, CA, USA). AlCl_3_·6H_2_O, DCFDA, dimethyl sulfoxide, calcium chloride, CH_3_COOK, dl-dithiothreitol, FeCl_2_, FeCl_3_, 2-thiobarbituric acid, 3-(2-pyridyl)-5,6-diphenyl-1,2,3-triazine-4′,4″-disulfonic acid sodium salt, deoxyribose, sodium nitrite, trichloroacetic acid (TCA), and other reagents were obtained from Sigma-Aldrich (St. Louis, MO, USA). The polyvinylidene fluoride (PVDF) membrane was purchased from Amersham Pharmacia Biotech Inc., Piscataway, NJ, USA. All other agents used in this study were of a reagent grade.

### 4.2. Antioxidant Capability Measurement

#### 4.2.1. Reducing Power Assay

The reducing power of CAE was measured using a slightly modified version of a previously described method [50]. Briefly, various concentrations of CAE (5–500 μg/mL) in 0.2 M PBS containing 1% ferrocyanate were prepared and then incubated for 20 min. Subsequently, the mixture was centrifuged after the addition of 10% TCA. The absorbance was measured at 700 nm after 1% ferric chloride solution was mixed with the supernatant. The absorbance intensity served as a proxy measure for the reducing power of the extract, as follows:

$$\mathrm{Reducing}\;\mathrm{power}(\%)=\left(\frac{A_{\mathrm{control}\;\mathrm{at}\;700\;\mathrm{nm}}-A_{\mathrm{sample}\;\mathrm{at}\;700\;\mathrm{nm}}}{A_{\mathrm{control}\;\mathrm{at}\;700\;\mathrm{nm}}}\right)\times 100$$

#### 4.2.2. Ferrous Ion Chelating Activity

The chelating of ferrous ions by CAE was assayed using a slightly modified version of the ferrozine assay employed in a previous study [50,51]. Briefly, 50–500 μg/mL CAE was added to 2 mM FeCl_2_ solution. After the addition of 5 mM ferrozine, the reaction was initiated. The absorbance was then spectrophotometrically determined at 562 nm by using a microplate reader (BioTek, Winooski, VT, USA). The results were calculated using the following equation and expressed as the percentage inhibition of the generation of the ferrozine-Fe^2+^ complex:

$$\mathrm{Chelating}\;\mathrm{effect}(\%)=\left(\frac{A_{\mathrm{control}\;\mathrm{at}\;562\;\mathrm{nm}}-A_{\mathrm{sample}\;\mathrm{at}\;562\;\mathrm{nm}}}{A_{\mathrm{control}\;\mathrm{at}\;562\;\mathrm{nm}}}\right)\times 100$$

#### 4.2.3. Hydroxyl Radical Scavenging Activity Assay

The capacity of CAE to scavenge hydroxyl radicals was estimated using a slightly modified version of a previously reported method [50,51]. The assay was performed by mixing 10–1000 μg/mL CAE, 10 mM ascorbic acid, 10 mM deoxyribose, 5 mM FeCl_3_, 1 mM EDTA, 10 mM H_2_O_2_, 65 μL KH_2_PO_4_-KOH buffer, and distilled water, and was then incubated for 15 min at 100 °C. A pink chromogen was formed and the mixture was centrifuged. Mannitol was used as the positive control. The absorbance of the supernatant was measured at 532 nm by using a microplate reader (BioTek, Winooski, VT, USA). The scavenging activity of CAE was obtained as the percentage inhibition of deoxyribose degradation, as follows:

$$\mathrm{Inhibition}(\%)=\left(\frac{A_{\mathrm{control}\;\mathrm{at}\;532\;\mathrm{nm}}-A_{\mathrm{sample}\;\mathrm{at}\;532\;\mathrm{nm}}}{A_{\mathrm{control}\;\mathrm{at}\;532\;\mathrm{nm}}}\right)\times 100$$

#### 4.2.4. Determination of Superoxide Anion Scavenging Activity

The ability of CAE to scavenge superoxide anions was assayed using a modified version of a previously reported model [50]. Reaction mixtures containing 936 μM dihydronicotinamide-adenine dinucleotide, 120 μM phenazine methosulfate, and 200 μM nitroblue tetrazolium were prepared in 0.1 M PBS, and CAE solution (1–50 μg/mL) was added. The mixture was incubated for 5 min. The absorbance was determined using an enzyme-linked immunosorbent assay reader (ELISA) (Tecan, Grödig, Austria) at 560 nm.

#### 4.2.5. Determination of Peroxide Scavenging Activity

The capability of CAE to scavenge H_2_O_2_ was spectrophotometrically measured by using a method described in a previous study [50,52]. H_2_O_2_ (20 mM) was prepared in PBS and added to various concentrations of CAE (50–1000 μg/mL). The mixture was incubated for 10 min. The absorption was detected using an ELISA reader (Tecan, Grödig, Austria) at 230 nm. The H_2_O_2_ scavenging activity of CAE was calculated using the following equation:

$$\mathrm{Scavenging}\;\mathrm{effect}(\%)=\left(\frac{A_{\mathrm{control}\;\mathrm{at}\;230\;\mathrm{nm}}-A_{\mathrm{sample}\;\mathrm{at}\;230\;\mathrm{nm}}}{A_{\mathrm{control}\;\mathrm{at}\;230\;\mathrm{nm}}}\right)\times 100$$

### 4.3. Cell Culture and UV Exposure

Hs68 cells were cultured and exposed to UV irradiation as previously described [53]. Cells were incubated in DMEM without serum in the presence of CAE for 24 h after UVB irradiation. A UV lighter (peak emission was 302 nm, CL-1000 M, UVP, Upland, CA, USA) was used for UVB exposure. UVB irradiation doses were 40–80 mJ/cm^2^ (exposure time was 15–30 s) [32,54].

### 4.4. Antioxidant Activity of CAE in Human Skin Fibroblasts

#### 4.4.1. Fluorescence Assay for Intracellular ROS in Fibroblasts

The fluorescence assay was performed using a slightly modified version of a previously described method [55]. Fibroblasts were planted for 24 h and then exposed to UVB light. The UVB irradiation dose was 80 mJ/cm^2^ (approximately 30 s). After incubation with CAE (1–50 μg/mL) for 2 h, the cells were incubated with 10 μM DCFDA in DMEM for 30 min. A fluorescence microscope was used to observe the images (Leica DMIL, Wetzlar, Germany), and the fluorescence (emission wavelength was 520 nm and excitation wavelength was 488 nm) was detected using an ELISA reader (Thermo Electron Corporation, Vantaa, Finland).

$$\mathrm{Relative}\;\mathrm{fluorescence}(\%)=\left(\frac{A_{\mathrm{control}}-A_{\mathrm{sample}}}{A_{\mathrm{control}}}\right)\times 100$$

#### 4.4.2. Catalase Activity Assay

Cells were incubated with CAE (5–50 μg/mL) for 24 h after UVB irradiation. Cells were harvested and centrifuged (1000× *g* at 4 °C for 10 min). The cell pellet was homogenized in 1 mL cold buffer (50 mM phosphate containing 1 mM EDTA, pH 6–7). The CAT content in the supernatant was measured using a CAT assay kit (Cayman Chemical Company, Ann Arbor, MI, USA). Briefly, 20 μL of the sample, 30 μL of methanol, and 100 μL of CAT assay buffer were added to a microplate, and the mixture was incubated on the plate in the dark on an orbital shaker for 20 min. Thirty μL of potassium hydroxide was added to terminate the reaction and the chromogen system, and a microplate reader (Tecan, Grödig, Austria) was used to measure the absorbance at 540 nm.

### 4.5. Immunoblot Analysis

Cells were incubated with CAE (5–50 μg/mL) for 24 h after UVB irradiation. Cells were collected and homogenized with lysis buffer as described in previous studies [53,54]. Equal protein amounts (30 μg) were loaded and separated on sodium dodecyl sulfate polyacrylamide gels and then transferred to a PVDF membrane. The membrane was incubated with specific antibodies, which comprised antibodies against IκB, p-IκB, and COX-2 (Santa Cruz Biotechnology, Inc., Santa Cruz, CA, USA). The blots were then incubated with antiimmunoglobulin G-horseradish peroxidase, and the ECL™ detection reagent (Amersham Biosciences, Buckinghamshire, UK) was added. Immunoreactive proteins were detected using an ECL Western blotting detection system (LAS-4000, Fujifilm, Tokyo, Japan), and density of bands was determined using a densitometric program (Multi Gauge V2.2, Fujifilm, Tokyo, Japan).

### 4.6. Immunofluorescence Staining

Cells were cultivated on glass cover slips as described in a previous paper [53]. After UVB exposure, various concentrations of CAE (5–25 μg/mL) were added and incubated for 24 h. The cells were then immersed in 4% paraformaldehyde and incubated with nonfat milk containing Triton X-100. A primary antibody was added to the cells, and the cells were then incubated with an Alexa Fluor 488 antirabbit IgG secondary antibody (Invitrogen, Carlsbad, CA, USA). The cells were washed with PBS buffer to rinse off the unbound secondary antibody. Finally, the samples were counterstained with the ProLong^®^ Gold antifade reagent and viewed using a microscope (Leica DMIL, Wetzlar, Germany).

### 4.7. Antiphotoaging Activity of CAE in the Hairless Mouse Skin

#### 4.7.1. Animals

Five-week-old BALB/cAnN.Cg-Foxn1^nu^/CrlNarl hairless female mice were obtained from the National Laboratory Animal Center in Taiwan. Animals were housed in individually controlled, ventilated caging systems with a relative humidity (50% ± 10%), controlled temperature (22 ± 2 °C), and 12-h light/dark cycle. The experimental protocols (100-124-N) were approved by the Institutional Animal Use and Care Committee of China Medical University (28 December 2010).

#### 4.7.2. UVB Irradiation and Topical Application of CAE

The hairless mice were randomly divided into five groups (six animals in each group): control, UVB treated, UVB treated with 50 μL vehicle, UVB exposed with 100 μg/mL CAE, and UVB exposed with 200 μg/mL CAE. The hairless mice in the UVB-exposed group were irradiated for 10 weeks on the dorsal skin, and the UV dose was similar to that described in a previous study [26,54]. In the vehicle group, 50 μL of glycerol was topically applied on the dorsal skin of the mice every day, and in the CAE group, glycerol containing 100 or 200 μg/mL CAE was applied every day. At the end of the 10-week experiment, the mice were sacrificed by an overdose of CO_2_. The dorsal skin specimens were immersed in formaldehyde. The skin slides were stained with Masson’s trichrome or hematoxylin and eosin, mounted on a coverslip, and observed under an optical microscope.

#### 4.7.3. Immunohistological Analysis of MMP-1, IL-6, and NF-κB

The sections of the hairless mouse skin were incubated with primary antibodies against MMP-1, IL-6, and NF-κB overnight. The sections were incubated with the secondary antibody after washing. The results were visualized under an optical microscope.

### 4.8. Statistical Analysis

All results are presented as the mean ± standard deviation of at least three independent experiments. *p* < 0.05 was considered statistically significant, and one-way analysis of variance, followed by a Scheffe’s test, was used to analyze the results.

## 5. Conclusions

**Figure 21:**
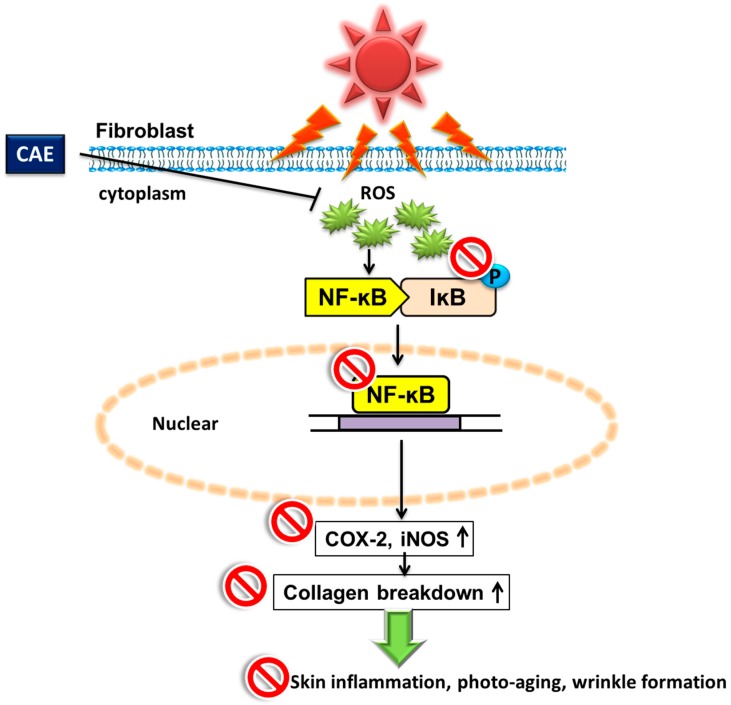
Scheme of CAE inhibition of UVB-induced inflammation and photodamage. (**↑**: upregulation; **─┤**inhibition; red circles: inhibition)

In this study, CAE exhibited potent antioxidant activity, including a high reducing capability, free radical scavenging capability, and high metal chelating activity, ameliorating UVB-induced ROS and enhancing CAT activity in human skin fibroblasts. In addition, CAE exhibited antiinflammatory activity, inhibited UVB-induced COX-2 and *p*-IκB expression, and suppressed NF-κB translocation to the nucleus (Figure 21). Finally, CAE reduced UVB-induced skin erythema and thickness, as well as increased the collagen content of the dermis in hairless mice. The results suggest that CAE can be useful for ameliorating aging- and photoaging-related disorders because of its antioxidant and antiinflammatory properties.

## Abbreviations

BHT	Dibutyl hydroxy toluene
CAE	*Coffea arabica* extract
CAT	Catalase
DCFDA	2′,7′-dichlorofluorescin diacetate
DMEM	Dulbecco’s modified Eagle’s medium
FBS	Fetal bovine serum
IL-6	Interleukin 6
NF-κB	Nuclear factor-kappa B
ROS	Reactive oxygen species
UV	Ultraviolet
